# Antenatal depressive symptoms in Jamaica associated with limited perceived partner and other social support: A cross-sectional study

**DOI:** 10.1371/journal.pone.0194338

**Published:** 2018-03-19

**Authors:** Omotayo Bernard, Roger C. Gibson, Affette McCaw-Binns, Jody Reece, Charlene Coore-Desai, Sydonnie Shakespeare-Pellington, Maureen Samms-Vaughan

**Affiliations:** 1 Department of Community Health & Psychiatry, The University of the West Indies, Mona, Kingston 7, Jamaica; 2 Department of Child & Adolescent Health, The University of the West Indies, Mona, Kingston 7, Jamaica; TNO, NETHERLANDS

## Abstract

**Background:**

Antenatal depression is associated with adverse maternal and infant well-being. However, compared to postpartum depression, it has been less frequently explored globally or in Jamaica. This study aimed to determine the prevalence of, and factors associated with, antenatal depressive symptoms among Jamaican women in order to inform policy and build interventions that could improve their management and reduce their negative consequences.

**Methods:**

This secondary analysis of data from the second Jamaican Birth Cohort Study (JA-Kids Birth Cohort) included 3,517 women enrolled during pregnancy. Information was extracted from interviewer-administered questionnaires which recorded social, demographic, medical and obstetric information during pregnancy. The Edinburgh Postnatal Depression scale (EPDS) was used to screen for depression, with scores ≥13 considered indicative of a high likelihood of depression. Bivariate analysis examined associations between depressive symptoms and: age, income, financial difficulties, perceived social support, perceived partner infidelity, previous child-bearing unions and children with the current partner. Obstetric factors were also explored and included gravidity, prior adverse pregnancy outcome and complications from previous pregnancies. Variables that predicted the likelihood of depression based on an EPDS cut score of 13 were evaluated using logistic regression.

**Results:**

One in five participants (19.6%; 95% CI 18.3–20.9%) had a high likelihood of antenatal depression (EPDS ≥13). Significant predictors of high depressive symptom severity included four indicators of poor perceived social and partner support [ORs (95% CI) ranged from: 1.61 (1.07–2.43); p = 0.024 to 3.14(1.69–5.84); p< 0.001], perceived partner infidelity [1.86 (1.36, 2.54); p<0.001], exposure to violence [2.36 (1.66–3.38); p<0.001] and financial difficulties [1.39 (1.07, 1.80); p = 0.013].

**Conclusions:**

Women’s perceived social and partner support were strongly associated with depressive symptom severity. Within the Jamaican cultural context of unstable reproductive unions, efforts are needed to involve fathers in the antenatal care process to strategically improve the psychological well-being of new mothers which may positively influence long term developmental outcomes for their babies.

## Introduction

One fifth to one third of women in developing countries will have mental health concerns associated with child-bearing [1]. Of these concerns, depressive symptoms are particularly prevalent [2, 3]. Worldwide, extensive research has been conducted on postpartum depression but antenatal depression has been less frequently explored [4]. In developing countries there is a general paucity of research on maternal mental health [2], and the number of studies on antenatal depression from Jamaica, a middle-income developing country, is also limited. Data from Pelotas, Brazil indicated that 16% (95%CI:14·9–17·1) of women screened using the Edinburgh Postnatal Depression Scale (EPDS) reported significant depressive symptoms (EPDS score ≥ 13) during the antenatal period [5]. Redinger and colleagues [6] found that in Soweto, South Africa 27% [95%CI: 24.2–29.8] of mothers had high EPDS (≥13) scores during the first trimester of pregnancy. Two previous Jamaican studies of antenatal depressive symptoms were confined to urban settings. The first used the Zung self-rating depression scale to screen a small sample (n = 78) of women at 28 weeks gestation. The researchers found that more than half of the women had depressive symptoms ranging from mild (31.3%) to moderate or marked (17.8%), to severe or extreme (6.9%)[7]. In the second study, Pottinger et al [8] assessed 452 women over each trimester of pregnancy using the EPDS and found that 25% had a probable depressive disorder (EPDS ≥13) over the three trimesters of pregnancy. Poverty, young age and inadequate social support were consistently reported by both studies as risk factors.

Studies which have highlighted an association between socio-demographic factors and antenatal depression [2, 4, 9] have reported that more women from low and middle income countries (LMICs) were predisposed to depressive symptoms [1, 2]. Contributing factors include inadequate income [2], financial difficulties [2], intimate partner violence [10], history of depression [8], and young maternal age [2, 9]. Young mothers are dually exposed given their limited access to financial resources while becoming pregnant without having supportive unions [9]. Inadequate partner support is an independent risk factor for depressive symptoms in the antenatal period [9, 11] while high levels of partner support have been shown to contribute to emotional well-being [12–14].

The family and partner relationship structures in Jamaican households are associated with different levels of social support. The three most common relationship patterns in Jamaica are the legally married union; the common-law union, with partners who live under the same roof but are not legally married; and the visiting relationship in which partners visit each other but reside in separate households [15]. While the visiting relationship is the most common it is also the most unstable, often resulting in child-bearing then separation and subsequently single parenting [16].

It is commonplace in Jamaica and the Caribbean for women to, over time, enter a number of different visiting or common-law relationships, one of which may result in marriage much later in life. Women thus often bear children for many different partners through their reproductive years. While this situation is associated with a perceived financial advantage based on the expectation of multiple sources of financial support from the different fathers of their children [17], in reality, this does not necessarily result in better socioeconomic and social support for these women. The current or new partner is sometimes hesitant to fully support the mother and her children, especially those not fathered by him, despite legal obligations to do so [15]. This uncertainty may put these mothers at risk for depressive symptoms. The possible association between antenatal depressive symptoms and childbearing for multiple partners has not previously been examined; nor has the issue of whether having more than one child with the current partner is an indicator of stability and therefore a potential protective factor against antenatal depression.

Another possible indicator of the stability of a relationship is the presence or absence of infidelity. Whisman, Gordon and Chatav [18] found that male spouses/partners were more likely to be unfaithful if they were dissatisfied in their relationship and their partners were also pregnant. Despite this, very little research has been conducted on the effect of partner infidelity on maternal depressive symptoms. One qualitative study highlighted that partner infidelity and partner rejection were possible causes of adverse maternal mental health [19], while Fisher et al. [2] reported that mothers in polygamous relationships had more symptoms of non-psychotic mental illnesses.

The stability of a partner relationship may also be influenced by its quality. Intimate partner violence (IPV) may undermine the quality, and therefore stability, of a relationship. Up to one third of Caribbean women report experiencing violence within intimate relationships with the risk of IPV being more frequent in common-law unions [20]. In a meta-analysis, the odds of high levels of depressive and anxiety symptoms in the antenatal period increased after exposure to IPV (OR 4.4, 95% CI 2.9–6.5) [21]. Women exposed to physical abuse during pregnancy have shown more emotional distress [22], with about one-third of pregnant women who experienced IPV having mental health concerns [23].

In addition to social and partner-related issues, obstetric factors may also be associated with antenatal depression. Significant obstetric variables have included multi-parity [24], past and current obstetric complications [25], medical comorbidities [26], previous pregnancy losses and unplanned pregnancy [27, 28].

Consequences of antenatal depression for mother and her offspring. Depression in pregnancy has been associated with adverse outcomes for the mother, child and family [29, 30]. Depressed mothers are less likely to access antenatal care [31], are more at risk for substance use, and are less likely to be astute about their nutritional needs and care required in pregnancy [32]. Antenatal depressive symptoms also increase the risk of postpartum depression [33, 34].

In both high and low income countries, antenatal depression has been associated with inadequate foetal nutrition with infants more likely to be born preterm, of low birthweight, or small for gestational age [35, 36]. Maternal depressive symptoms in the second and third trimesters of pregnancy have been associated with poorer mother-infant bonding 8 weeks postnatally [37]. Over the long term, infants born to mothers with antenatal depressive symptoms are more likely to exhibit impaired growth (low weight for height, low height for age) [38] and behavioural difficulties [39, 40]. Anxiety disorders have even been evident in offspring at 18 years of age [41].

In South Africa, high levels of depressive symptoms (EPDS>13) were associated with HIV seropositivity, food insecurity and alcohol use [42]. Remarkably, these women and their children benefitted from social interventions which had a positive impact on the children’s cognitive and physical development. Further national level research in Jamaica could therefore help to inform appropriate policies and interventions to improve the management of antenatal depression and reduce its associated negative outcomes. Against the background of challenges with family stability in the Jamaican setting as well as an inadequate understanding of maternal mental health concerns, this study aimed to determine the extent to which Jamaican pregnant women experienced depressive symptoms. We also sought to explore possible associations of these symptoms with demographic, social and partner related issues (maternal age, income, financial difficulties, perceived social support, partner infidelity, previous child-bearing unions, and previous children with the current partner) while controlling for possible confounding obstetric parameters (gravidity, previous adverse pregnancy outcomes, and complications from previous pregnancies).

## Methods

**Fig 1:**
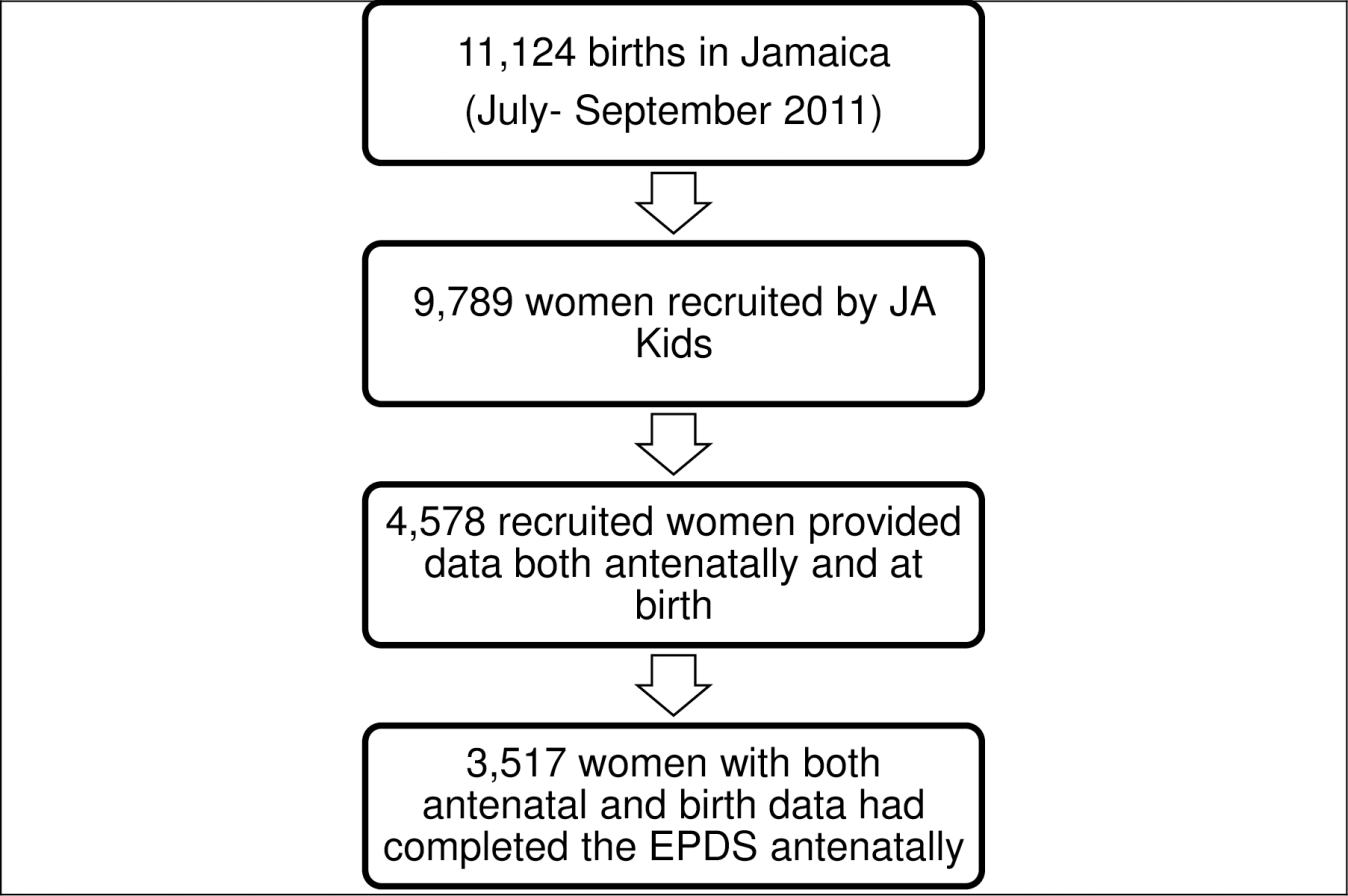
Acquisition of data on participants.

Secondary analyses were conducted on data collected during the antenatal period in the second Jamaican Birth Cohort Study (JA Kids; (https://www.mona.uwi.edu/fms/jakids/ This was a nationwide survey which incorporated 87% of women who gave birth in all regions of the country from July 1- September 30, 2011. The goal of JA Kids was to update our understanding of issues affecting Jamaican families, especially children. Women were recruited throughout the antenatal period from public and private sector antenatal clinics as well as at birth. Women eligible for this study were (a) those recruited during the antenatal period (47% of the 9789 recruited participants), (b) responding to the mental health screening questions, and (c) providing information at delivery (n-3517, 36%) (See Fig 1). The study satisfied the requirements of the Declaration of Helsinki and was approved by the ethics committees of Jamaica’s Ministry of Health (approval #198; 2011) and the University of the West Indies (approval # ECP 122 10/11). All participants provided written informed consent.

### Instruments

All questionnaires were administered in person by interviewers who were trained in recruitment and consent procedures, interview techniques, a standardized approach to applying the questionnaires, and the maintenance of participant confidentiality. They were competent to offer clarifications and explanations to participants when requested. Women were free to refuse to answer questions they considered too personal. Women recruited during pregnancy answered three interviewer administered questionnaires on (a) My Expectations of My Pregnancy, My Parenting and My Partner; (b) My Life, My Home, My Community; and (c) My Health. The first (168 items) included information on partner relationships and expectations. The second (211 items) explored social and family characteristics as well as details about the home environment. The third questionnaire (219 items) inquired about the mothers’ reproductive history, health and well-being prior to the pregnancy. The Edinburgh Postnatal Depression scale (EPDS) was embedded in the third questionnaire. Initially developed by Cox, Holden and Sagovsky [43] to screen for postpartum depression among women in the community, it has also been widely used for screening in the antenatal population [44, 45]. A systematic review of validation studies among antenatal women showed that cut-off EPDS scores ranging from 5 to 15 yielded sensitivities of 64–100% and specificities of 73–100% [45]. The current study utilized EPDS score categories from the 2015 South Australian Perinatal Practice Guidelines [46] and other published work [47, 48]. Summary scores of 0–9 were considered to represent a low likelihood; 10–12 a moderate likelihood; and ≥13 a high likelihood of depression.

### Variables of interest

Given the research objectives, variables selected for analysis from the JAKIDS database included the EPDS score, socio-demographic measures (age, gravidity, income and financial difficulty), parameters pertaining to perceived social support and partner related issues, exposure to violence and previous obstetric conditions.

### Data analysis

*Perceived social support* was explored by considering the partner’s reaction (supportive, indifferent or resentful) to the pregnancy at its initial diagnosis and at the time of the interview, along with other social support indicators (perceived frequency of available love and affection; emotional support and help with chores). No composite social support index was created, and each variable was considered independently. *Partner-related issues* examined relationship stability and included perceived partner fidelity, the number of children for the current partner and the number of previous childbearing unions. Partner fidelity was examined using women’s responses to a question regarding whether they thought their partner had any other sexual contacts in the year before the pregnancy. The only measure of *exposure to violence* was one item on “physical abuse within the last year.” Data were not available on intimate partner violence (IPV).

Table 1 details the available obstetric history variables, their prevalence and the response rates for the study. For the bivariate analyses the presence of any of the listed adverse pregnancy outcomes (e.g. miscarriage, termination, stillbirth, early or late/post neonatal death) were combined to create a variable “any adverse outcome” while a positive response to any of the listed obstetric complications (e.g. preterm labour, premature rupture of membranes, antepartum or postpartum haemorrhage, hypertension in pregnancy, pre-eclampsia, eclampsia, HIV disease, diabetes in pregnancy), were used to create the variable “any prior pregnancy complication”.

**Table 1:** History of previous adverse pregnancy outcomes and previous obstetric complications among multi-gravid women recruited during pregnancy.

Previous history of the following conditions	Women recruited during pregnancy (n, %)	
Total	2717	
ADVERSE PREGNANCY OUTCOMES	N	%
Miscarriages	655	31.2
- **Response rate**	(2099, *77*)	
Terminations of pregnancy	200	9.9
- **Response rate**	(2019, *74*)	
Still births	83	4.2
- **Response rate**	(1970, *73*)	
Live births dying within one week of birth	48	2.5
- **Response rate**	(1946, *72*)	
Live births dying later than first week of birth	43	2.2
- **Response rate**	(1942, *71*)	
Premature births (<37 weeks)	113	5.8
- **Response rate**	(1959, *72*)	
Babies with weight < 2,500g	194	9.9
- **Response rate**	(1963, *72*)	
PAST OBSTETRIC COMPLICATIONS		
Caesarean section	267	10.5
- **Response rate**	(2553, *94*)	
Forceps delivery	25	1.0
- **Response rate**	(2526, *93*)	
Pelvic pain	97	3.8
- **Response rate**	(2541, *94*)	
Early labour (<37 weeks)	143	5.6
- **Response rate**	(2553, *94*)	
Premature rupture of membranes	83	3.3
- **Response rate**	(2547, *94*)	
Antepartum haemorrhage	121	4.7
- **Response rate**	(2552, *94*)	
Post partum haemorrhage	230	9.0
- **Response rate**	(2547, *94*)	
Thrombosis	17	0.7
- **Response rate**	(2544, *94*)	
Pulmonary embolism	1	0.04
- **Response rate**	(2544, *94*)	
Bacterial infections	270	10.7
- **Response rate**	(2552, *94*)	
HIV	7	0.3
- **Response rate**	(2532, *93*)	
Diabetes in pregnancy	42	1.7
- **Response rate**	(2542, *94*)	
Pre-eclampsia or toxaemia	27	1.1
- **Response rate**	(2535, *93*)	
Seizures	17	0.7
- **Response rate**	(2538, *93*)	
Hyperemesis	307	12.0
- **Response rate**	(2550, *94*)	

#### Statistical analysis

In order to avoid having cells with too few participants, some variables were collapsed into broader groups. The income groups were combined into three categories (low, middle and high) representing the closest approximation to tertiles from the ten mutually exclusive household income bands. The tertiles corresponded to household incomes equivalent to US$ 0–250, 251–425 and >425 per month. Medians and interquartile ranges were determined for continuous variables (non-normal distributions were demonstrated in preliminary analyses). Frequencies and percentages were determined for the categorical variables. The chi square test was applied to explore relationships between categorical variables, using SPSS version 18. Statistical significance was taken at p< 0.05.

Finally a multivariate logistic regression analysis was performed using high likelihood of depression (EPDS score of ≥13) as the dependent variable. To simplify the interpretation of the multivariate analysis, some categorical covariates with more than two categories were converted into binary variables. Variables with the four options of “none,” “a little,” “some,” or “most” were grouped into “none or minimal” and “some or most.” These include the variables: available love and affection, help with chores and emotional support. For partners’ perceived initial and current reactions to the pregnancy, the “supportive” category was retained and the “indifferent” and “resentful” categories were combined to form a single new category of “not supportive.” For financial difficulty, the “some” and “great” categories were grouped together as “present” while the “none” category was re-labelled as “absent.” Given the high prevalence of missing data (31.6%), for the variable “partner infidelity,” these cases (n = 1111) were included as a third response category in the multivariate model. Covariates included were those that were significantly associated with the EPDS score from the bivariate analysis. Variables initially entered in the regression model were systematically removed if they were not statistically significant contributors (p>0.05) until only covariates significantly associated with the outcome variable (p< 0.05) were retained. Listwise deletion was used for missing data.

## Results

Table 2 compares sociodemographic characteristics of all women in the cohort with those recruited during pregnancy and with the subset of those recruited during pregnancy for whom EPDS scores were available. There were no differences in age or gravidity among the three groups.

**Table 2:** Characteristics of the women in the JA Kids cohort.

Characteristics of study participants	Entire Ja Kids Maternal Cohort		Women recruited during pregnancy		Women recruited during pregnancy with available EPDS scores	
	N	%	N	%	N	%
**Total participants**	9789		4578		3517	
**Maternal age**						
**<20 years**	1853	19.2	933	21.1	721	21.0
**20–34 years**	6514	67.4	2955	66.7	2307	67.3
**35+ years**	1297	13.4	541	12.2	401	11.7
**Total/ Missing**	9664/125	98.7/1.3	4429/149	96.7/3.3	3429/88	97.5/2.5
**Gravidity/ Current pregnancy**						
**-First**	3856	39.4	1703	38.5	1311	38.1
**-Second**	1606	16.4	1083	24.5	864	25.1
**-Third or more**	4320	44.2	1634	37.0	1263	36.8
**Total/ Missing**	9782/7	99.9/0.1	4420/158	96.5/3.5	3438/79	97.8/2.2

The median gestational age when the EPDS was administered was 28 (IQR 11) weeks, suggesting that when interviewed, women and their partners already had some time to adapt to the pregnancy and the imminent birth. Only 1.3% of interviews were conducted in the first trimester; 46% in the second, and 53% in the third. The median maternal age (IQR) was 24 (10) years with 21% of participants being adolescents (<20 years) and 12% women who were 35 years and older. Median gravidity (IQR) was 2 (2), with 39% of women being pregnant for the first time and 37% experiencing their third or higher order pregnancy. (Table 2). Most multi-gravid mothers reported no previous obstetric complications (61.3%; n = 1445) or adverse pregnancy outcomes (61.5%; n = 1160).

Table 3 compares economic, obstetric, social and partner related issues between all antenatal recruits and those with EPDS scores. No differences were noted for these characteristics between the two populations. Most EPDS respondents fell in the low income category (57%) with 17% reporting relatively high income. While 42% did not report any financial difficulties, 47% reported some difficulties and 10% stated that they had great difficulties; this latter group was overrepresented by low income mothers (χ^2^_4_ = 75.01, p <0.01). While understandable that two of three (66%) women of low income were having some or great financial difficulties, in the high income group as many as 47% had similar financial challenges.

**Table 3:** Economic, obstetric, social support and partner related factors among women recruited during pregnancy and those in the current study with available EPDS scores.

Characteristics of study participants	Women recruited during pregnancy		Women recruited during pregnancy with available EPDS scores	
	N	%	N	%
**Total**	4578		3517	
**ECONOMIC PARAMETERS**				
**Monthly household income (USD)**				
**-low ($0–250)**	1469	56.0	1155	56.9
**-middle ($251–425)**	692	26.4	528	26.0
**-high ($>425)**	461	17.6	346	17.1
**Total/ Missing**	2622/2316	57.3/42.7	3029/488	86.1/13.9
**Financial difficulty**				
**-none**	1608	42.4	1249	42.4
**-some**	1799	47.4	1379	46.8
**-great**	388	10.2	318	10.8
**Total/ Missing**	3795/783	82.9/17.1	2946/571	83.8/16.2
**OBSTETRIC PARAMETERS**				
**Previous adverse pregnancy outcomes**				
**Absent**	2863	79.8	2445	79.6
**Present**	726	20.2	576	20.4
**Total/Missing**	3589/989	78.4/21.6	3021/496	85.9/14.1
**Previous obstetric complications**				
**Absent**	3148	77.5	2440	77.1
**Present**	912	22.5	725	22.9
**Total/Missing**	4060/518	88.7/11.3	3165/352	90.0/10.0
**PERCEIVED SOCIAL SUPPORT**				
**Partner’s initial reaction**				
**-resentful**	153	3.5	120	3.6
**-indifferent**	340	7.9	275	8.2
**-supportive**	3836	88.6	2945	88.2
**Total/ Missing**	4329/249	94.6/5.4	3340/177	95.0/5.0
**Partner’s current reaction**				
**-resentful**	139	3.2	110	3.3
**-indifferent**	230	5.3	185	5.5
**-supportive**	3989	91.5	3056	91.2
**Total/ Missing**	4358/220	95.2/4.8	3351/166	95.3/4.7
**Available love and affection**				
**-none of the time**	128	3.2	102	3.3
**-a little of the time**	684	17.3	537	17.4
**-some of the time**	1161	29.4	870	28.1
**-most of the time**	1979	50.1	1585	51.2
**Total/ Missing**	3952/626	86.3/13.7	3094/423	88.0/12.0
**Available help with chores**				
**-none of the time**	204	5.5	165	5.7
**-a little of the time**	1305	35.4	1016	35.3
**-some of the time**	1014	27.5	765	26.6
**-most of the time**	1167	31.6	931	32.4
**Total/ Missing**	3690/888	80.6/19.4	2877/640	81.8/18.2
**Available emotional support**				
**-none of the time**	134	3.4	105	3.4
**-a little of the time**	964	24.7	746	24.4
**-some of the time**	1176	30.1	901	29.5
**-most of the time**	1636	41.8	1305	42.7
**Total/ Missing**	3910/668	85.4/14.6	3057/460	86.9/13.1
**PARTNER RELATED ISSUES**				
**Partner infidelity**				
**-present**	963	30.9	767	31.9
**-absent**	2154	69.1	1639	68.1
**Total/ Missing**	3117/1461	68.1/31.9	2406/1111	68.4/31.6
**Number of children with current partner**				
**- 0**	2973	68.8	2317	69.0
**- 1–2**	1178	27.3	912	27.1
**- ≥ 3**	170	3.9	131	3.9
**Total/ Missing**	4321/257	94.4/5.6	33607/158	95.5/4.5
**Number of previous child-bearing unions**				
**- 0**	2258	52.4	1746	52.0
**- 1–3**	1979	46.0	1554	46.3
**- ≥ 4**	69	1.6	57	1.7
**Total/ Missing**	4306/272	94.1/5.9	3357/160	95.5/4.5
**EXPOSURE TO VIOLENCE**				
**-present**	486	10.6	393	11.3
**-absent**	3956	86.4	3077	88.7
**Total/ Missing**	4442/136	97.0/3.0	3470/47	98.7/1.3

Primigravidae included among persons with “absent” obstetric findings

### Partner related issues and exposure to violence

Overall, most mothers reported consistent partner support from the initial phases of pregnancy (89%) through to the time of the interview (92%). The proportion of men who were not keen to have a child (resentful) remained the same when told initially (3.6%) as well as midway through the pregnancy (3.2%). More men who were initially indifferent (7.92%) warmed to the idea, however, with the proportion declining to 5.3% over the two periods. Other available specific forms of perceived social support (not necessarily from the women’s partners) were high with 80% of women reporting available love and affection some or most of the time. The corresponding figures were, however, somewhat lower for emotional support (72%) and help with chores (59%). Of the 3117 (68%) women who responded to the question regarding partner infidelity, as many as 31% of study participants thought their partners had been unfaithful during the year leading up to pregnancy. Just 11% of women reported exposure to physical violence. (Table 3).

Most women provided information on both their childbearing experience with the current partner and previous childbearing unions (95%). For 69%, this was the first child with the man who fathered the current pregnancy; however 46% reported one to three previous child bearing unions and 1.6% (69) had four or more children for other partners. (Table 3)

### EPDS score and socio-demographic characteristics

One in five mothers (19.6%; 95% CI: 18.3%-20.9%; n = 690) were assessed as having a high likelihood of antenatal depression (EPDS ≥13) while another 14.4% had a moderate likelihood of depression (scores 10–12). EPDS score categories were not significantly associated with maternal age (χ^2^_4_ = 7.10, p = 0.131), income (χ^2^_4_ = 8.33, p = 0.08), gravidity (χ^2^_4_ = 1.54, p = 0.820), previous adverse pregnancy outcome (χ^2^_2_ = 2.28, p = 0.32), number of previous child-bearing unions (χ^2^_4_ = 4.56, p = 0.33), or number of children for the current partner (χ^2^_4_ = 2.11, p = 0.72). (Table 4).

**Table 4:** Socio-demographic and obstetric parameters of antenatal women and EPDS score.

Variable, Number of respondents (n) and response rates (%)	Variable categories	EPDS Categories (Risk of Depression)		
		Low Likelihood	Moderate Likelihood	High Likelihood
		(scores ≤9)	(scores 10–12)	(scores ≥13)
		n (%)	n (%)	n (%)
**TOTAL**	All respondents	2320 (66.0)	507 (14.4)	690 (19.6)
**Age (years)**	<20	472 (65.5)	97 (13.5)	152 (21.0)
4429 (*97*)	20–34	1529 (66.3)	320 (13.9)	458 (19.8)
	35+	269 (67.1)	69 (17.2)	63 (15.7)
**Gravidity/ Current**	First	876 (66.8)	181 (13.8)	254 (19.4)
**pregnancy**	Second	936 (66.1)	202 (14.3)	278 (19.6)
4420 (*97*)	Third or greater	457 (64.3)	110 (15.5)	144 (20.3)
Past obstetric complication^*^^1^	Absent	1658 (68.0)	328 (13.4)	454 (18.6)
4060 (*89*)	Present	442 (61.0)	126 (17.4)	157 (21.7)
**Previous adverse pregnancy outcome**	Absent	1519 (67.7)	310 (13.8)	416 (18.5)
3589 (*78*)	Present	381 (66.1)	73 (12.7)	122 (21.2)
**Income**	Low	779 (67.5)	153 (13.2)	223 (19.3)
2622 (*57*)	Middle	375 (71.0)	63 (12.0)	90 (17.0)
	High	236 (68.2)	59 (17.1)	51 (14.7)
Financial difficulty^*^^2^	None	918 (73.5)	153 (12.2)	178 (14.3)
3795 (*83*)	Some	881 (63.9)	219 (15.9)	279 (20.2)
	Great	170 (53.5)	48 (15.1)	100 (31.4)
**Number of previous**	0	1171 (67.1)	241 (13.8)	334 (19.1)
**child-bearing unions**	1–3	1021 (65.7)	224 (14.4)	309 (19.9)
4306 (*94*)	≥4	31 (54.4)	12 (21.1)	14 (24.6)
**Number of children with**	0	1540 (66.5)	315 (13.6)	462 (19.9)
**current partner**	1–2	605 (66.3)	136 (14.9)	171 (18.8)
4321 (*94*)	≥3	84 (64.1)	22 (16.8)	25 (19.1)
Partner infidelity*^3^	Absent	1190 (72.6)	221 (13.5)	228 (13.9)
3117 (*68*)	Present	403 (52.5)	134 (17.5)	230 (30.0)
**Exposure to violence**	Absent	2134 (69.4)	419 (13.6)	524 (17.0)
4442 (*97*)*^4^	Present	163 (41.5)	79 (20.1)	151 (38.4)

*Statistically significant associations at p<0.05 based on chi square test.

^1^ χ^2^_2_ = 12.84, p = 0.002

^2^ χ^2^_4_ = 66.89, p< 0.001

^3^ χ^2^_2_ = 108.33, p< 00.01

^4^ χ^2^_4_ = 133.11, p< 0.001

However, mothers who reported financial difficulties were more likely to have high EPDS scores (31% with great difficulties versus 14% with no difficulties) (χ^2^_4_ = 66.89, p< 0.001). Similarly, those mothers who experienced previous obstetric complications were disproportionately found among women with the highest EPDS scores (χ^2^_2_ = 12.84, p = 0.002). The 32% of women who thought their partners had been unfaithful also had higher levels of depressive symptoms (χ^2^_2_ = 108.33, p<0.001) as did women who reported exposure to physical violence (χ^2^_2_ = 133.11, p<0.001). (Table 4).

### Association of perceived social support, exposure to violence and partner issues with depressive symptoms

All variables assessing perceived social support were significantly associated with EPDS score categories with women reporting lower levels of perceived social support having a higher likelihood of depression. (Table 5).

**Table 5:** Antenatal women’s perceptions of social support and EPDS scores.

		EPDS Score Categories (Risk of Depression)		
Perceived Social Support Parameters and response rates n (%)				
		Low	Moderate	High
		Likelihood	Likelihood	Likelihood
		(scores ≤9)	(scores 10–12)	(scores ≥13)
		n (%)	n (%)	n (%)
Partner’s Initial	Resentful	53 (44.2)	18 (15.0)	49 (40.8)
Reaction*^1^	Indifferent	127 (46.2)	52 (18.9)	96 (34.9)
**4329 (*95*)**	Supportive	2052 (69.7)	407 (13.8)	486 (16.5)
Partner’s Current	Resentful	36 (32.7)	15 (13.6)	59 (53.6)
Reaction*^2^	Indifferent	71 (38.4)	43 (23.2)	71 (38.4)
**4358 (*95*)**	Supportive	2130 (69.7)	420 (13.7)	506 (16.6)
Available Love & Affection*^3^	None of the time	43 (42.2)	17 (16.7)	42 (41.2)
	A little of the time	267 (49.7)	99 (18.4)	171 (31.8)
**3952 (*86*)**	Some of the time	557 (64.0)	145 (16.7)	168 (19.3)
	Most of the time	1193 (75.3)	185 (11.7)	207 (13.1)
Available Help with Chores*^4^	None of the time	81 (49.1)	32 (19.4)	52 (31.5)
	A little of the time	646 (63.6)	134 (13.2)	236 (23.2)
**3690 (*81*)**	Some of time	516 (67.5)	119 (15.6)	130 (17.0)
	Most of the time	727 (78.1)	103 (11.1)	101 (10.8)
Available Emotional Support*^5^	None of the time	47 (44.8)	11 (10.5)	47 (44.8)
	Little of the time	394 (52.8)	137 (18.4)	215 (28.8)
**3910 (*85*)**	Some of time	617 (68.5)	144 (16.0)	140 (15.5)
	Most of the time	997 (76.4)	141 (10.8)	167 (12.8)

*Statistically significant associations at p<0.05 based on chi square test.

^1^ χ^2^_4_ = 111.8, p< 0.001

^2^ χ^2^_4_ = 172.2, p< 0.001

^3^ χ^2^_6_ = 171.1, p< 0.001

^4^ χ^2^_6_ = 95.0, p< 0.001

^5^ χ^2^_6_ = 178.9, p< 0.001.

### Regression analysis

From the bivariate analyses, the covariates initially entered into the logical regression model were financial difficulties, previous obstetric complications, available love and affection, available help with chores, available emotional support, partner’s initial reaction, partner’s current reaction, partner infidelity and exposure to violence. Love and affection and previous obstetric complications were not retained. The final model (Table 6) included seven independent predictors of a high likelihood of antenatal depression; four related to perceived social support, one socio-economic and two other partner related indicators. Women who said they received little emotional support [OR(95%CI) = 3.14 (1.69–5.84); p< 0.001] were three times more likely than others to have high EPDS scores. Also at risk for high EPDS scores were women who reported having little help with chores [OR(95%CI) = 1.83 (1.12–3.00); p = 0.016]. This was similar for women who reported that their partners were not keen on a new baby either initially [OR(95%CI) = 1.61 (1.07–2.43); p = 0.024] or at the time of the interview [OR(95%CI) = 2.68 (1.71–4.20); p< 0.001]. Women who expressed that their partners had been unfaithful [OR(95%CI) = 1.86 (1.36–2.54); p< 0.001] or were reluctant to respond [OR(95%CI) = 1.54 (1.14–2.08); p = 0.005] and those exposed to physical violence [OR(95%CI) = 2.36 (1.66–3.38); p< 0.001] were also at high risk. Finally, women who reported having financial difficulties were 39% more likely to report high levels of depressive symptoms [OR(95%CI) = 1.39 (1.07–1.80); p = 0.013].

**Table 6:** Logistic regression model of predictors of EPDS score (< 13 or ≥13) with sociodemographic and perceived social support indicators, perceived partner infidelity, and exposure to violence as independent co-variates (n = 1972).

Independent covariates	Response rate	Odds ratio	p value
	n (%)	(95% CI)	
**Sociodemographic indicators**			
Financial difficulties	3795 (*83*)		
-Absent		1	
-Present		1.39 (1.07, 1.80)	0.013
**Perceived Social Support**			
Frequency of Available help with chores	3690 (*81*)		
-Some or most of the time		1	
-None or minimal		1.83 (1.12, 3.00)	0.016
Frequency of Emotional Support	3910 (*85*)		
-Some or most of the time		1	
-None or minimal		3.14 (1.69, 5.84)	<0.001
Partner’s Initial Reaction	4329 (*95*)		
-Supportive		1	
-Not Supportive		1.61 (1.07, 2.43)	0.024
Partner’s Current Reaction	4538 (*95*)		
-Supportive		1	
-Not Supportive		2.68 (1.71, 4.20)	<0.001
**Perceived partner Infidelity**	3117 (*68*)		
-Absent		1	
-Present		1.86 (1.36, 2.54)	<0.01
-Undisclosed/ missing		1.54 (1.14, 2.08)	0.005
**Exposure to physical violence**	4442 (*97*)		
-Absent		1	
-Present		2.36 (1.66, 3.38)	<0.001

## Discussion

One in five antenatal women had a high likelihood of depression. This is similar to both the 17.9% rate of moderate to severe depressive symptoms documented from a smaller Jamaican sample by Wissart et al. [7], and the 25% rate of probable depressive disorder reported among another Jamaican sample which used the same EPDS cut score as the current study [8]. These three Jamaican studies provide a fair amount of evidence that there is a significant risk of antenatal depression among Jamaican women. Of note the current study is larger and more inclusive of women from all social strata, compared to the previous two.

### Perceived social support

Positive perceptions of social support were protective against depressive symptoms, and while the majority of participants reported some perceived social support those with deficiencies in this area were at greater risk for depression. The most important element of perceived emotional support was the general “availability of emotional support.” Two partner-specific components that were significant were his initial and current reaction to the pregnancy. Strategies that strengthen pregnant women’s social, and especially emotional, support are likely to be beneficial to them as are methods of enhancing their partners’ level of positivity towards the pregnancy.

Of interest is the lack of significance of obstetric factors which were excluded from the final regression model. These observations suggest that interventions for antenatal women at risk for depression should focus on social support as advocated by Reblin and Uchino [49], especially given the relatively high incidence of childbearing in unstable unions among Jamaican women. To this end, new strategies, e.g. participation in support groups, as well as pre-existing social resources, e.g. partners and families, may be utilized. Within this context, further research may help to determine the value of specific interventions, such as the inclusion of partners in routine antenatal visits and methods for highlighting their roles as partners and parents. It would also be worthwhile to consider developing clinical strategies which integrate screening and referral for risk of depression into routine antenatal care, with intervention strategies varying by symptom severity.

### Other partner related issues

Contrary to the expectations that a higher likelihood of depression would be associated with both a high number of child bearing partners and women having their first pregnancy with their current partner, neither association was found. Dreher and Hudgins [16] highlighted that multiple child bearing unions could be a beneficial adaptive strategy employed by the Jamaican mothers of lower socioeconomic backgrounds. This study does not contradict that perspective.

Walcott and Hickling [50] recently highlighted that Jamaican men reported difficulty in sustaining a monogamous relationship and were likely to have several partners. Partner infidelity affects relationship security and may result in relationship discord, depression and issues of low self-esteem [51]. Mothers who are dissatisfied with or anxious about the security of their relationship have been reported to be at increased risk of distress [52]. This is compounded by the fact that partners of pregnant women are more likely to engage in multiple dyadic relationships in comparison to partners of non-pregnant women [18]. One previous study found a marginal association between partner infidelity and depressive symptoms, with affected mothers more likely to record a moderate to high likelihood of depression [53].

### Exposure to violence

Exposure to violence was associated with depressive symptoms among mothers in this cohort, consistent with that of many other researchers [54, 55], despite inadequate availability of data to explore intimate partner violence.

### Financial difficulty

Financial difficulty but not income was associated with antenatal EPDS scores, suggesting that income may not be a good indicator of financial well-being in this population. Indeed, almost half of women in the high income category reported some or great financial difficulty, suggesting that a lifestyle component may be contributory. Besides income, other possible determinants of financial well-being might include support from other sources such as the extended family and revenues from abroad. Another consideration is that some mothers may not have adequately declared their income. The association between financial difficulty and antenatal depression has previously been observed [1, 9, 56].

### Interplay of risk and protective factors

The issues that have been found to be associated with a heightened likelihood of antenatal depression cannot be considered independently of each other. For any given pregnant woman, there is likely to exist an interplay of risk and protective factors. For example, a woman who is in an unstable relationship where her unborn child is not welcomed by the father and he has been having other intimate relationships is likely to be particularly vulnerable to depression. This effect would be compounded if she were also unemployed and not receiving adequate financial support from her partner. On the other hand, if she had good emotional support from her family of origin, this might offset some of the tendency towards depression. Unfortunately, for many women the risk factors may far outweigh the protective ones. Such women would benefit from even more intensive efforts to reduce their risk of antenatal depression and its attendant adverse outcomes.

### Limitations

Because this was a secondary analysis of existing data, the study’s instruments were not designed to optimally address the research objectives. For example, the baseline of maternal psychiatric disorder and previous experiences of pregnancy related depressive symptoms were not determined. Thus, a distinction could not be made between pre-existing and pregnancy related depressive symptoms. The cross-sectional nature of the study imposed another limitation. Although depressive symptoms may vary over time during a woman’s pregnancy, the assessment was restricted to a single point in time in this study. The face-to-face nature of the interview may also have resulted in the under-reporting of symptoms if women were uncomfortable about sharing very personal concerns with an interviewer. Thus the reported prevalence rates could be lower than the actual rates.

The use of the EPDS would have been strengthened had it been validated for the assessment of antenatal depression in Jamaica. Nevertheless, its validation for the assessment of antenatal depression in both high and low income settings elsewhere [45, 57, 58], provides some justification for use in the current study. The instrument has also been widely used by other investigators in a number of LMIC settings including Jamaica [8], Brazil [5] and South Africa [6, 42]. The same EPDS cut score of 13 that was used in the current study has also been employed by these investigators.

Missing data may have affected the strength or accuracy of some results obtained. Although participants with missing data were excluded only from the analyses for which the required data item were unavailable, it is possible that the reasons, though unknown, for which data were missing could have biased the findings. The highest levels of missing data were for income (57% response rate), a variable which the Jamaican population is not keen to divulge. For this reason, the variable assessing “financial difficulties” was included. Women were asked to qualitatively evaluate how well they were coping with meeting their financial needs and more women (83%) responded to this item. That women in the highest income tertile also reported challenges, is an indication that despite income, the day to day demands of life may increase with social mobility and may outstrip available income.

Women were less willing to discuss issues of partner fidelity (68% response rate). The very personal nature of these issues may have made some participants reluctant to answer the relevant questions. Including non-responders in the regression model documented that silence is somewhat suggestive of concerns that persons are unwilling to articulate. Such issues are a challenge when conducting large scale studies but hopefully this approach to handling significant missing data can address some of the concerns regarding potential bias. We acknowledge that alternative methods of dealing with missing data, e.g. regression imputation, could have been applied.

While less than 5% of Jamaican women fail to attend for antenatal care [59], the possible exclusion of women with poor health seeking behaviour may be of concern especially when assessing mental health indicators. Weighting was not considered as the study sample was drawn from a clearly defined population of women who did not differ remarkably from the base population. This consisted of all women who gave birth from July to September 2011 from all regions nation-wide, 87% of whom agreed to participate either during pregnancy or at the time of delivery.

### Conclusions and recommendations

A substantial proportion of Jamaican antenatal women had a moderate (14%) or high likelihood (20%) of depression with poor perceived social and partner support being strongly associated with a high likelihood of depression. Other significant associated factors were financial difficulty, partner infidelity and exposure to violence. Complications of pregnancy were not significant. The salience of the association with perceived social and partner support is suggestive of there being value in establishing or enhancing strategies that seek to strengthen social resources. This could include involving partners and families more in standard antenatal care, sensitizing them to the value of their support, and discussing practical ways in which their support may be realized. This will however require policy changes in how antenatal care services are delivered and may benefit from small field trials to test the effectiveness of specific interventions prior to their incorporation in routine practice [42]. Greater awareness by the clinical team of the high prevalence of the problem could facilitate the integration of routine screening with the EPDS, after appropriate clinical validation for use in this setting. The presence of any of the factors that were associated with a higher likelihood of depression should also alert clinicians to the need for further mental health evaluation and/ or intervention.

## Supporting information

S1 Dataset Data on cohort of 4,578 Jamaican women recruited during pregnancy. (SAV)
